# Factors Predicting Malignancy in Patients with Polymyositis and Dermatomyostis: A Systematic Review and Meta-Analysis

**DOI:** 10.1371/journal.pone.0094128

**Published:** 2014-04-08

**Authors:** Xin Lu, Hanbo Yang, Xiaoming Shu, Fang Chen, Yinli Zhang, Sigong Zhang, Qinglin Peng, Xiaolan Tian, Guochun Wang

**Affiliations:** China-Japan Friendship Hospital, Beijing, China; Keio University School of Medicine, Japan

## Abstract

**Objective:**

To define potential factors that could predict concomitant neoplastic diseases in patients diagnosed with PM/DM, which could inform screening decisions.

**Methods:**

Two researchers independently reviewed articles from Pubmed (MEDLINE), EMBASE, Cochrane Plus Library and ISI Web of Knowledge with no restrictions on study design or language. Given that some of the studies combined PM and DM patients as research subjects while others included only DM patients, data were subjected to meta-analyses for all combined PM/DM studies and studies that included only DM patients to obtain informative results.

**Results:**

For PM/DM patients, the following factors are all associated with an increased risk of malignancy: older age, age greater than 45, male sex, dysphagia, cutaneous necrosis, cutaneous vasculitis, rapid onset of myostis (<4 weeks), elevated CK, higher ESR, higher CRP levels. Several factors were associated with lower-than-average risk, including the presence of ILD, arthritis/arthralgia, Raynaud's syndrome, or anti-Jo-1 antibody. For DM patients, results indicated an increased risk of malignancy with older age, male sex, the presence of cutaneous necrosis, elevated ESR (>35 mm/hr), higher CRP levels, or anti-p155 antibody. In addition, the presence of anti-ENA antibodies seem to be related to reduced risk of malignancy.

**Conclusion:**

Awareness and implementation of early-stage cancer screening in PM/DM patients who have these identified factors – such as being older than 45, male sex, cutaneous necrosis, cutaneous vasculitis – are of crucial importance from public health and clinical perspectives and provide insight into the etiopathogenesis of CAM.

## Introduction

Idiopathic inflammatory myopathies (IIM) are a group of acquired, heterogeneous systemic diseases that mainly affect skeletal muscle. The main IIM subtypes include polymyositis (PM), dermatomyositis (DM) and inclusion-body myositis (IBM). Many epidemiological studies have been conducted to substantiate the association between IIM and malignancy. The overall malignancy risk in these patients is higher than that in the age- and sex-matched general population. This elevated risk is particularly pronounced in IIM patients within three years of their initial diagnosis[1], [2]. The comorbid frequency of malignancy in IIM was reported to range from 3% to 40%[3], [4]. Each subtype of IIM has been reported to have an association with malignancy, including PM, DM and IBM[1], [2], [5]. Of the three IIM subtypes, DM appears to have the strongest association with malignancy. According to reports, adenocarcinoma was the most common type of IIM-related cancer[2], [6]. Additionally, certain cancers – e.g. ovarian, lung, breast and pancreatic cancer in patients with DM, and lymphatic and hematopoietic malignancies such as non-Hodgkin's disease in patients with PM – were over-represented compared to the general population in western countries, specifically in Europe and north America[7]. In contrast, nasopharyngeal cancer has long been reported as the predominant cancer associated with DM in many Asian countries, including Hong Kong, Singapore and Taiwan[8].

IIM patients who suffer from malignancy have poorer prognoses than those without malignancies. Therefore, early identification of IIM patients with a high risk of developing malignancies would benefit their survival. Much research has been published describing demographic, clinical and laboratory factors associated with malignancy in patients with IIM. However, these studies had certain limitations, such as small sample sizes, inconsistent inclusion of factors, and results that were more controversial than conclusive. Consequently, it is difficult for clinicians to determine the extent of investigations necessary to test for the presence of malignancy at the onset of myositis as well as the necessary frequency/intensity of repeat testing.

The purpose of this systematic review and meta-analysis was to determine which factors increase the risk for malignancy in IIM patients and to estimate the level of risk heightened by each factor in relation to average-risk IIM patients. Few studies have incorporated IBM as an inclusion criterion when selecting predictors for IIM-associated malignancy. Thus, this study synthesized all available evidence to examine potential predictors for malignancy in PM/DM patients.

## Methods

### Data sources and searches

An extensive electronic literature search was conducted before September 2013 on four international databases of scientific literature (MEDLINE, EMBASE, Cochrane Plus Library, ISI Web of Knowledge). The search strategy used the medical subject heading (MeSH) terms “polymyositis” OR “dermatomyositis” OR “myositis” OR “inflammatory myopathy” combined with synonyms of malignancy. No language restrictions were applied. References lists of relevant papers were screened. We also searched abstracts from conferences organized by the American College of Rheumatology(ACR), the European League against Rheumatism(EULAR), and the Asia Pacific League of Associations for Rheumatology(APLAR). This systematic review was planned, conducted and reported in adherence to the developed guidelines for reporting meta-analysis[9].

### Study selections

Our study included all relevant articles-including randomized, controlled trials; prospective or retrospective cohort studies; nested case-control studies or population-based case-control studies that reported at least one demographic, clinical or laboratory factor and compared them with cases that did and did not have associated malignancy. We included studies that provided sufficient information to allow the calculation of risk ratios (RRs), weighted mean differences (WMD) and standardized mean differences (SMD) for the risk of developing malignancy in PM/DM patients. Factors reported as having significant association with malignancy in PM/DM in at least one article were considered potential predictors and were included in our study. We excluded studies with less than 20 subjects so that the included studies would have sufficient potential to be examined for any possible association between at least one potential predicting factor and malignancy. When we encountered studies that were conducted from one group on similar subjects, only the higher-quality study was included. Studies were excluded if they did not include a control group for comparison or if they provided data by combining PM, DM and IBM together.

### Data extraction and quality assessment

The articles were independently reviewed by two researchers. Preliminary review covered journal titles and abstracts. Articles were selected if either researcher felt they were relevant or if neither researcher could determine whether they were irrelevant. The full text of the articles selected were then reviewed by applying predetermined inclusion and exclusion criteria. Additional articles were included after manually reviewing reference lists of papers that met inclusion criteria. From the included studies, an investigator extracted the following data: first author, year of publication, study design, characteristics of study population, number of subjects enrolled, enrollment criteria, duration of follow-up, predicting factor, outcome ascertainment, analytic methods (including adjustment for confounders), and associations between predictor variables and malignancies in patients with PM and DM, including relevant statistics. A second investigator examined the extracted data for accuracy. In addition, the quality of the nonrandomized studies was assessed by using the Newcastle-Ottawa Scale (NOS). In all cases, disagreement among the reviewers was resolved through discussion until a consensus was obtained. To retrieve any missing data, we also contacted the authors of the primary studies.

### Data synthesis and analysis

Meta-analysis of the results was conducted when results could be obtained from at least two studies. Given that some of the studies were conducted on a combination of PM and DM patients together and that others included only DM patients, data were subjected to meta-analyses for all combination studies and studies that included only DM patients to obtain informative results.

For dichotomous variables, meta-analysis of RRs – which were weighted estimates of risk factors – was performed. For continuous variables, meta-analysis of the differences between means was performed. When subgroups had to be combined for meta-analysis, Higgins and Green formulas[10] were used to obtain the means and joint standard deviations. When the studies reported medians and ranges instead of means and standard deviations, the means and standard deviations were calculated according to methods described by Hozo SP et al[11]. WMDs and corresponding 95% confidence intervals (CIs) were calculated from the raw data of the selected studies. However, when the units of the outcomes in the original articles were inconsistent, or when mean differences were too large, SMDs were used as the summary statistic. SMD thresholds were defined as negligible (<0.2), small (0.2–0.6), moderate (0.6–1.2), and large (>1.2)[12]. We assessed the statistical heterogeneity of the studies by using standard chi-square tests, and we measured the magnitude of heterogeneity by using the I^2^ statistic. An I^2^ value greater than 50% was considered to indicate significant heterogeneity, and an I^2^ value less than 25% was considered to indicate no significant heterogeneity[13]. We used a random-effects model to account for variation among the studies. In general, when there was no variation among studies, the random-effects model yielded the same results as a fixed-effects model without a study effect[14]. To explore heterogeneity, we used meta-regression analysis to assess the impact of the proportions of DM patients included in the original studies, study location (Western countries vs. Asian countries) and sample sizes (>100 vs. ≤100). However, meta-regression analysis was especially inefficient when the number of studies included was small[15]. Therefore, analysis of the subgroups that were stratified by the study characteristics mentioned above was another option, if permitted. We also conducted sensitivity analyses by repeating the original analyses while separately omitting one study at a time to assess whether any single study had a disproportionately large influence on the results. We looked for evidence of publication bias by applying Egger's test to funnel plots of RRs when results from five or more studies were pooled[16]. If the funnel plot was asymmetrical, we adjusted by applying the “trim and fill” method[17]. All analyses were performed by using Stata, version 11.0 (StataCorp, College Station, Texas).

## Results

**Figure 1:**
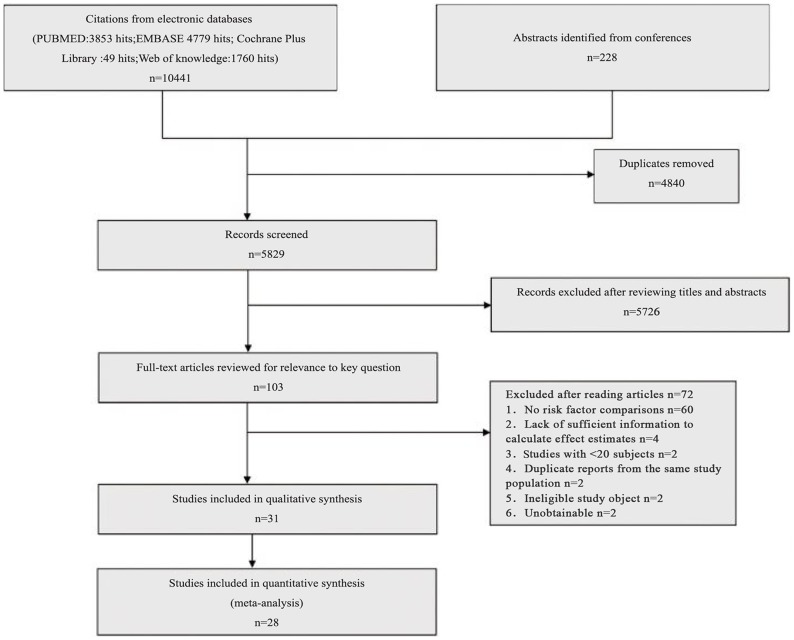
Flow diagram of the study selection process.

**Figure 2:**
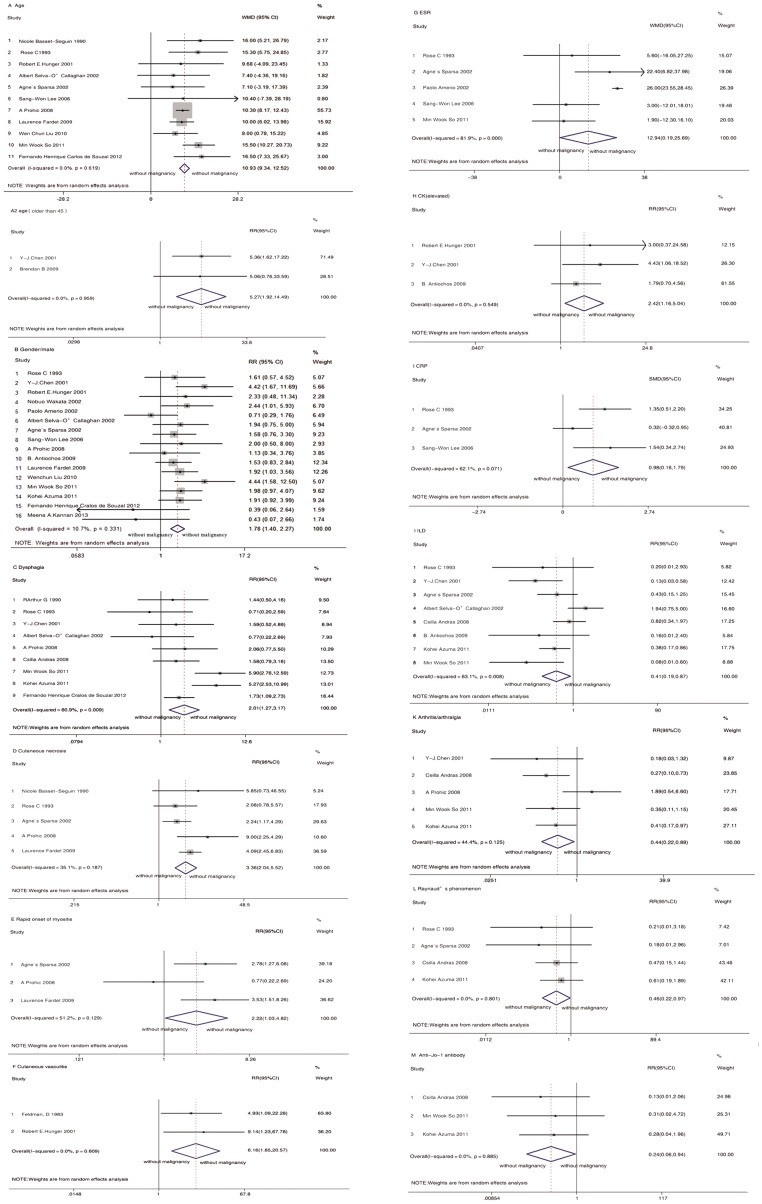
Forest plots generated by random-effects meta-analysis for the significant findings from all studies. (A) Age. (B) Gender. (C) Dysphagia. (D) Cutaneous necrosis. (E) Rapid onest of myositis. (F) Cutaneous vasculitis. (G) ESR. (H) CK(elevated). (I) CRP. (J) ILD. (K) Arthritis/arthralgia. (L) Raynaud phenomenon. (M) Anti-Jo-1 antibody.

**Figure 3:**
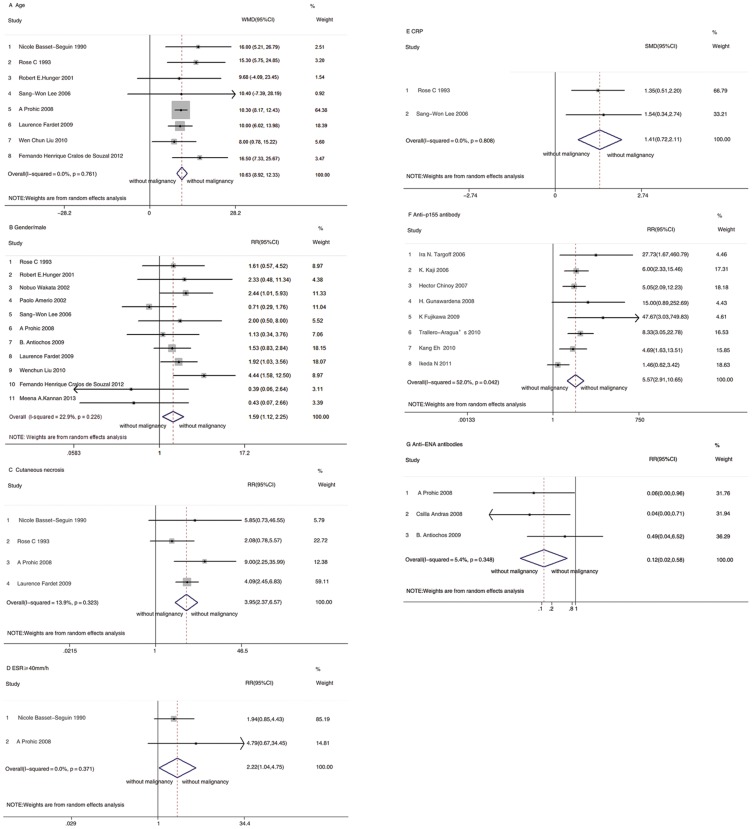
Forest plots generated by random-effects meta-analysis for the significant findings from studies involving DM patients only. (A) Age. (B) Gender. (C) Cutaneous necrosis. (D) ESR(≥40 mm/h). (E) CRP. (F) Anti-p155 antibody. (G) Anti-ENA antibodies.

A total of 10441 potential primary studies were detected in the MEDLINE, EMBASE, Cochrane Plus Library and ISI Web of Knowledge databases. This number was reduced to 5829 following the elimination of duplicates. All titles and abstracts were screened, and 101 papers were identified as having potential for further evaluation. After reviewing the full text of the articles, 28 studies of 22 PM/DM-associated malignancy predictors were included in the final analysis.(diagrammed in Figure 1) In total, data from 27 respective cohorts studies, and one[18] prospective cohorts studies were included in the final analysis. Out of these 28 studies, 21 of these studies included only DM patients, and seven studied both PM and DM patients. Study characteristics are listed in Table S1, online only, and ratings of study quality for each of the Newcastle-Ottawa Scale (NOS) criteria are presented in Table S2, online only. Figure 2 to 3 present forest plots generated by random-effects meta-analysis for the significant findings from all studies and studies involving DM patients only respectively. Sensitivity analyses were conducted, indicated the original results were robust.

### Demographic Variables

Two demographic variables of age (n = 815) and gender (n = 1171) were included in the final analysis.

Of the included studies, 13 studies[19], [20], [21], [22], [23], [24], [25], [26], [27], [28], [29], [30], [31] considered age as a variable. Out of these 13 studies, two studies[29], [30] provided age group-specific data: data for patients younger than 45 and data for patients 45 and older. Data from other studies were presented as continuous data. A meta-analysis of 11 studies[19], [20], [21], [22], [23], [24], [25], [26], [27], [28], [31] indicated that malignancy associated with PM/DM was associated with older age (WMD 10.93, 95% CI: 9.34 to 12.52). For patients with DM only,[19], [20], [21], [22], [23], [24], [25], [31] a similar difference was observed in data pooled from the eight relevant studies (WMD 10.63, 95% CI: 8.92 to 12.33). As noted, two estimates[29], [30] from two studies of PM/DM patients divided the subjects according to age by using “45” as the age divider. This division of subjects contributed to the meta-analysis with an overall RR of 5.27 (95% CI: 1.92 to 14.49).

A total of 16 studies[20], [21], [22], [23], [24], provided data on gender and included a total of 1171 patients (274 males, 23.40%). Findings from this analysis suggest that there was a significant association between male gender and malignancy associated with PM/DM. The RR for all studies was 1.78 (95% CI: 1.40 to 2.27). The estimate for the 11[20], [21], [22], [23], [24], [25], [30], [31],[32],[33],[35] of DM patients only was 1.59 (95%CI: 1.12 to 2.25).

### Clinical Variables

**N**ine clinical variables were identified in the final analysis: dysphagia (n = 832), cutaneous necrosis (n = 253), interstitial lung disease (ILD)(n = 727), arthritis/arthralgia (n = 557), fever (n = 308), Raynaud phenomenon (n = 297), periungual erythema (n = 298), rapid onset of myositis (n = 255), cutaneous vasculitis (n = 99).

Analysis of data from nine of the studies[20], [26], [27], [29], [31], [33], [34], [36], [37] showed that malignancy risk was significantly higher in PM/DM patients with dysphagia (RR, 2.00; 95% CI: 1.27 to 3.12). The estimate for the five studies[20], [27], [31], [36], [37] involving DM patients only was 1.24 (95% CI: 0.76 to 2.02).

A meta-analysis of the five studies[19], [20], [23], [24], [28] involving cutaneous necrosis indicated that PM/DM patients with cutaneous necrosis faced a significantly higher risk of malignancy (RR, 3.36; 95% CI: 2.04 to 5.52). For patients with DM only,[19], [20], [23], [24] a similar difference was observed in the pooled data of the four relevant studies (RR, 3.95; 95% CI: 2.37 to 6.57).

Judging from eight of the studies[20], [26], [27], [28], [29], [30], [34], [37], the risk of malignancy was significantly reduced in PM/DM patients who had ILD (RR,0.41; 95% CI: 0.19 to 0.87). But the estimate for the three studies[20], [30], [37] that included DM patients only was 0.54 (95% CI: 0.19 to 1.55).

From five of the studies,[24], [26], [29], [34], [37] we learned that the risk of malignancy was significantly reduced in PM/DM patients with arthritis/arthralgia (RR, 0.44; 95% CI: 0.22 to 0.89). The estimate for two studies[24], [37] that involved DM patients only was 0.69 (95% CI: 0.10 to 4.74).

Three studies [27], [29], [37] evaluated the effect of fever on the risk of malignancy in patients with PM/DM. Pooled data did not indicate that fever was a relevant factor (RR, 0.60; 95% CI: 0.14 to 2.60).

Four studies[20], [28], [34], [37] revealed that the risk of malignancy was significantly reduced in PM/DM patients who had Raynaud phenomenon (RR, 0.46; 95% CI: 0.22 to 0.97). The estimate for two studies that involved DM patients only was 0.42 (95% CI: 0.15 to 1.18).

Three studies[23], [24], [29] evaluated the influence of periungual erythema on the risk of malignancy in patients with PM/DM and found no association (RR, 1.51; 95% CI: 0.57 to 4.01). The estimated RR for two studies[23], [24] involving DM patients only was 2.25 (95% CI: 0.75 to 6.82).

Rapid onset of myositis – defined here by a diagnosis being made within four months of the appearance of initial symptoms – was investigated in three studies. Pooled data[23], [24], [28] indicated statistically significant increased risk for malignancy in PM/DM patients who experienced a rapid onset of myositis (RR, 2.22; 95% CI: 1.03 to 4.82). Two studies[23], reported the average time between the onset of symptoms and a DM diagnosis. Pooled data did not indicate that this time was any shorter in the group of that suffered malignancies (WMD, −2.17; 95% CI: −5.72 to 1.39).

Two studies[18], [21] provided estimates for the meta-analysis of PM/DM patients with cutaneous vasculitis compared to those without cutaneous vasculitis. Risk of malignancy was significantly increased in PM/DM patients who had cutaneous vasculitis (RR, 6.16; 95% CI: 1.85 to 20.57).

### Laboratory Variables

11 clinical variables were identified in the final analysis: erythrocyte sedimentation rate (ESR)(n = 459), creatine kinase (CK)(n = 968), albumin (n = 207), C-reactive protein (CRP)(n = 85), aspartate aminotransferase (AST)(n = 339), alanine aminotransferase (ALT)(n = 191), lactate dehydrogenase (LDH)(n = 479), antinuclear antibody (ANA)(n = 669), anti-extractable nuclear antigen (anti-ENA) antibodies (n = 213), anti-Jo-1 antibody (n = 349), anti-p155 antibody (n = 406).

Ten studies[19], [20], [22], [24], [25], [26], [28], [30], [33], [36] reported ESR from PM/DM patients with malignancies and those without. Out of these, six studies[19], [24], [25], [30], [33], [36] of DM patients presented the data as dichotomous outcomes, but the cut-off points (elevated, 25, 35 or 40 mm/hr) were too disparate to be combined as reported in the published studies. Therefore, we pooled data from studies that used the same cut-off point and presented the results of this single study in a descriptive and qualitative manner. The pooled data of two studies[19], [24] indicated that an ESR higher than 40 mm/hr was strongly associated with malignancy in patients with DM (RR, 2.22; 95% CI: 1.04 to 4.75). However, the pooled data of three studies,[25], [30], [36] which provided data comparing patients with elevated ESR levels and those with normal levels, indicated no increased risk of malignancy in DM patients (RR, 1.54; 95% CI: 0.84 to 2.82). Five studies[20], [22], [26], [28], [33] of PM/DM patients can be pooled to yield a WMD estimate of 12.94 (95% CI: 0.19 to 25.69), which indicated a higher level of ESR in the group that suffered malignancies. The estimate for three studies[20], [22], [33] involving DM patients only was 13.22 (95% CI: −4.85 to 31.28).

Data on CK levels, captured from 13 studies, were presented as continuous and dichotomous data. Summary SMD of CK levels[20], [22], [23], [24], [26], [28], [31], [33], [35], [37] was 0.11 (95% CI: −0.63 to 0.85), indicating no difference between the CK levels of the two groups. The estimate for eight studies[20], [22], [23], [24], [31], [33], [35], [37] involving DM patients only was 0.28 (95% CI: −0.69 to 1.24). Three studies[21], [29], [30] of PM/DM patients reported outcomes as elevated or not. Pooled data from these studies indicated statistically significant increased risk for malignancy in patients with elevated CK levels (RR, 2.42; 95% CI: 1.16 to 5.04).

Three studies[20], [22], [28] presented data on albumin levels in PM/DM patients, both those with malignancy and those without. Summary SMD of albumin levels between the two groups was −0.37 (95% CI: −1.09 to 0.35).

Three articles[20], [22], [28] evaluating CRP levels in PM/DM patients with malignancy and those without were included in the meta-analysis. Summary SMD was 0.98 (95% CI: 0.16 to 1.79). The estimate for two studies[20], [22] involving DM patients only was 1.41 (95% CI: 0.72 to 2.11).

Four studies[22], [23], [24], [26] evaluating AST levels in PM/DM patients with malignancy and those without were included in the meta-analysis. Summary SMD was 0.65 (95% CI: −0.61 to 1.91). The estimate for three studies[22], [23], [24] involving DM patients only was 1.14 (95% CI: −1.21 to 3.49).

Three studies[22], [23], [24] evaluating ALT levels in DM patients with malignancy and those without were included in the meta-analysis. Summary SMD was 0.79 (95% CI: −2.01 to 3.59).

Six studies[22], [23], [24], [26], [33], [37] evaluating LDH levels in PM/DM patients with malignancy and those without were included in the meta-analysis. Summary SMD was 0.12 (95% CI: −0.82 to 1.06). The estimate for five studies[22], [23], [24], [33], [37] involving DM patients only was 0.32 (95% CI: −0.90 to 1.54).

Eight studies[23], [24], [25], [26], [28], [30], [34], [37] evaluated the effect of ANA on the risk of malignancy in PM/DM patients. Pooled data indicated that ANA was not a relevant factor affecting malignancy risk (RR, 1.15; 95% CI: 0.79 to 1.68). The estimated RR for five studies[23], [24], [25], [30], [37] involving DM patients only was 0.95 (95% CI: 0.57 to 1.60).

In a meta-analysis of three studies[30], [37], [38], anti-ENA antibodies were associated with a reduced risk of malignancy in DM patients (RR, 0.12; 95% CI: 0.02 to 0.58).

In a meta-analysis of three studies[26], [34], [37], the anti-Jo-1 antibody was associated with a reduced risk of malignancy in PM/DM patients (RR, 0.24; 95% CI: 0.06 to 0.94).

In a meta-analysis of eight studies[6], [39], [40], [41], [42], [43], [44], [45], the anti-p155 antibody was associated with an increased risk of malignancy in DM patients (RR, 5.57; 95% CI: 2.91 to 10.65).

### Heterogeneity and Publication Bias

Table 1 provides the results of heterogeneity and Egger's test for each of the variables identified above. We performed meta-regression analysis and evaluated (1) study location (Western countries vs. Asian countries), (2) sample sizes (>100 vs. ≤100), and (3) different proportions of DM patients in original studies of the following variables that exhibit significant statistically heterogeneity: dysphagia, ILD, ESR, CK, AST, LDH. In univariate analysis, the aforementioned three study characteristics were found to be significant confounders for dysphagia. The coefficient associated with the proportion of DM patients was negative, and the coefficient for sample size was positive. Study location could explain some of the variations in ILD and ESR. Stratified analyses were conducted for these three variables (Table 2). Evidence of funnel plot asymmetry and other small study effects were observed when we analyzed anti-p155 antibody levels. Application of the “trim and fill” method yielded a corrected RR of 4.34 (95% CI: 2.32 to 8.12).

**Table 1:** Associations of PM/DM Associated Malignancy with Each Factors.

Factors	PM/DM*				DM only			
	RR/WMD/SMD[95%CI]	Heterogeneity		Egger's test(P)	RR/WMD/SMD[95%CI]	Heterogeneity		Egger's test(P)
		P	I^2^(%)			P	I^2^(%)	
Age	WMD:10.93 (9.34 to 12.52)	0.62	0.0	0.71	WMD:10.63 (8.92 to 12.33)	0.76	0.0	0.86
Age(≥45)	RR:5.27 (1.92 to 14.49)	0.96	0.0	NA	NA	NA	NA	NA
Gender(Male)	RR:1.78 (1.40 to 2.27)	0.33	10.7	0.58	RR:1.59 (1.12 to 2.25)	0.23	22.9	0.78
Dysphagia	RR:2.00 (1.27 to 3.12)	0.01	60.9	0.06	RR:1.48 (0.76 to 2.02)	0.62	0.0	NA
Cutaneous necrosis	RR:3.36 (2.04 to 5.52)	0.19	35.1	0.66	RR:3.95 (2.37 to 6.57)	0.32	13.9	NA
ILD	RR:0.41 (0.19 to 0.87)	0.01	63.1	0.14	RR:0.54 (0.19 to 1.55)	0.30	16.0	NA
Arthritis/arthralgia	RR:0.44 (0.22 to 0.89)	0.13	44.4	0.99	RR:0.69 (0.10 to 4.74)	0.02	82.9	NA
Fever	RR:0.60 (0.14 to 2.60)	0.08	59.8	NA	NA	NA	NA	NA
Raynaud phenomenon	RR:0.46 (0.22 to 0.97)	0.80	0.0	NA	RR:0.42 (0.15 to 1.18)	0.59	0.0	NA
Periungual erythema	RR:1.51 (0.57 to 4.0)	0.09	58.0	NA	RR:2.25 (0.75 to 6.82)	0.18	44.8	NA
Time△ (week)	WMD:−2.17 (−5.72 to 1.39)	0.59	0.0	NA	NA	NA	NA	NA
Rapid onset of myositis(≤4 week)	RR:2.22 (1.03 to 4.82)	0.13	51.2	NA	NA	NA	NA	NA
cutaneous vasculitis	RR:6.16 (1.85 to 20.57)	0.61	0.0	NA	NA	NA	NA	NA
ESR(≥40 mm/h)	NA	NA	NA	NA	RR:2.22 (1.04 to 4.75)	0.37	0.0	NA
ESR(≥35 mm/h)□	NA	NA	NA	NA	RR:34.94 (4.96 to 245.88)	NA	NA	NA
ESR(elevated)☆	NA	NA	NA	NA	RR:1.54 (0.84 to 2.82)	0.76	0.0	NA
ESR(mm/h)	WMD:12.94 (0.19 to 25.69)	0.00	81.9	0.06	WMD:13.22 (−4.85 to 31.28)	0.00	83.3	NA
CK	SMD:0.11 (−0.63 to 0.85)	0.00	93.2	0.26	SMD:0.28 (−0.69 to 1.24)	0.00	94.5	0.25
CK(elevated)☆	RR:2.42 (1.16 to 5.04))	0.00	0.55	NA	NA	NA	NA	NA
Albumin	SMD: −0.37 (−1.09 to 0.35)	0.02	75.0	NA	NA	NA	NA	NA
CRP	SMD:0.98 (0.16 to 1.79)	0.07	62.1	NA	SMD:1.41 (0.72 to 2.11)	0.81	0.0	NA
AST	SMD:0.65 (−0.61 to 1.91)	0.00	93.1	NA	SMD:1.14 (−1.21 to 3.49)	0.00	94.8	NA
ALT	NA	NA	NA	NA	SMD:0.79 (−2.01 to 3.59)	0.00	95.7	NA
LDH	SMD:0.12 (−0.82 to 1.06)	0.00	93.2	0.37	SMD:0.32 (−0.90 to 1.54)	0.00	94.3	0.48
ANA	RR:1.15 (0.79 to 1.68)	0.13	37.3	0.27	RR:0.95 (0.57 to 1.60)	0.15	41.1	0.48
Anti-ENA antibodies	NA	NA	NA	NA	RR:0.12 (0.02 to 0.58)	0.35	5.4	NA
Anti-Jo-1 antibody	RR:0.24 (0.06 to 0.94)	0.89	0.0	NA	NA	NA	NA	NA
Anti-p155 antibody	NA	NA	NA	NA	RR:5.57 (2.91 to 10.65)	0.04	52.0	0.04

PM  =  Polymyostis; DM  =  Dermatomyositis; ILD  =  Interstitial lung disease; ESR  =  Erythrocyte sedimentation rate; CK  =  Creatine kinase; CRP  =  C-reactive protein; AST  =  Aspartate aminotransferase; ALT  =  Alanine aminotransferase; LDH  =  Lactate dehydrogenase; ANA  =  Antinuclear antibody; ENA  =  extractable nuclear antigen; NA  =  not available.

*PM/DM: the results of all studies (studies that included PM and DM combined as research subjects and that included DM patients only).

△ Time between the appearance of initial symptoms and the diagnosis of PM/DM was made.

□ Only one study reported ESR using a cutoff point of 35.

☆ elevated: higher than normal level.

**Table 2:** The Results of Stratified Analyses.

Factor	Category	RR/WMD	95%CI	Heterogeneity	
				P	I^2^(%)
Dysphagia	Study location				
	Western populations	RR:1.53	1.12 to 2.10	0.65	0.0
	Asian populations	RR:4.11	2.06 to 8.21	0.14	49.4
	Sample size				
	>100	RR:2.67	1.46 to 4.88	0.02	69.2
	≤100	RR:1.25	0.72 to 2.19	0.50	0.0
ILD	Study location				
	Western populations	RR:0.70	0.31 to 1.59	0.18	42.1
	Asian populations	RR:0.21	0.08 to 0.56	0.20	38.7
ESR	Study location				
	Western populations	WMD:22.31	13.15 to 31.47	0.17	43.5
	Asian populations	WMD:2.42	−7.89 to 12.73	0.92	0.0

## Discussion

This meta-analysis summarizes the available evidence on potential predictors of malignancy in PM/DM patients. Of the included studies, 22 factors were observed for meta-analysis. For all studies, 19 factors – excluding AST, anti-ENA antibodies and the anti-p155 antibody – were analyzed. For studies involving DM patients only, 17 factors were analyzed (excluding five factors: fever, rapid onset of myositis, albumin, cutaneous vasculitis and anti-Jo-1 antibody). This information may be used to inform cancer screening and improve our understanding of how each of these factors contribute to malignancy.

### Factors associated with an increased risk of malignancy

Results indicate that PM/DM patients who are older than 45, suffer cutaneous necrosis, suffer cutaneous vasculitis, suffer dysphagia, experienced a rapid onset of myositis (<4 months between onset of initial symptoms and diagnosis) and elevated CK were at least twice as likely to develop malignancy. Being male was associated with a 1.5- to 2-fold increase in malignancy risk. In addition, higher ESR and higher CRP were observed in the group of malignancy. Owing to the different type of research subjects involved and possible indistinct relationships between PM/DM and malignancy, we conducted another analysis on studies involving DM patients only. These studies showed that DM patients with cutaneous necrosis, elevated ESR (>35 mm/hr) and the anti-p155 antibody faced at least a two-fold increase in risk for malignancy. Older age, male sex and higher CRP were traits more common in DM patients with malignancy than in those without. Therefore, PM/DM patients with these features should receive a comprehensive cancer screening, and this awareness should inform the follow-up consultations and treatments as well.

In this study, we compared different age groups of patients with and without associated malignancies and found that older age was significantly associated with malignancy in both PM and DM patients. Previous studies had reported that malignancy is very rare in pediatric patients. However, malignancies become more common in children with unusual conditions – such as atypical rash, splenomegaly, or impressive lymphadenopathy[46] – and extensive evaluation for malignancy at the time of diagnosis in these patients is crucial. As described above, adult dermatomyositis positive for anti-p155 were more likely to develop malignancy than negative patients and anti-p155 was the most frequent autoantibody in the juvenile idiopathic inflammatory myopathies[47], but it seems nothing to do with malignancy in children patients. Recently, Lisa G. Rider et al[47] undertook a large study to define the full spectrum of demographic, clinical features, and outcomes associated with the myositis autoantibodies in patients with the juvenile idiopathic inflammatory myopathies(JIIM), and found that children with anti-p155/140 had unique clinical features compared to adults. One limitation in the study they acknowledged was that they did not distinguish between TIF1γ and TIF1α,it is not difficult to stimulate our imagination that whether a possibly different antigen (not TIF1-γ) exist in this group of patients. If the same antigen of the two groups generating different phenotypes, it may show different pathogenetic mechanisms between JIIM and adult patients with myositis from another angle. Anti-p155 antibody can be served as a clue, which deserve our in-depth research in the future. The development of necrotic lesions in the context of DM is a rare occurrence, but the results of our meta-analysis reemphasised this clinical parameter, which can be easily identified by a dermatologist, is probably one of the most important indications for a detailed investigation for underlying cancer in DM. Two studies[18], [21] had reported that cutaneous vasculitis was more frequently observed in PM/DM patients with malignancy, even though it is not unusual in PM/DM. Typically, the vasculitis is non-necrotic. In a study of 60 patients with vasculitis associated with malignancy, Fain and colleagues[48] found that cutaneous leukocytoclastic vasculitis (LV) was the most frequent paraneoplastic type. These observations indicated that PM/DM and LV may concurrently be involved in the occurrence of paraneoplastic rheumatic syndrome and inspired oncologists and rheumatologists to investigate the relationship between these two diseases and malignancy. We found that PM/DM patients with malignancy had higher serum CRP and ESR levels than the patients without, especially for DM patients, whose ESR level was above 35 mm/h. Although the patient numbers studied in our study were small, this may give a tantalizing clue as to serum markers for predicting malignancy in PM/DM patients. Trallero-Araguas et al [49]did a systematic review and meta-analysis to quantify the usefulness of the anti-p155 antibody for cancer-associated myositis(CAM) prediction. This analysis included 312 adult patients with DM pooled from six studies. The diagnostic odds ratio was 27.26(95%CI: 6.59–112.82), which meant that positive testing to p155 had a 27.28 higher association with cancer than p155-negative status. In our study, two new studies were added and the RR value was 5.57(95%CI:2.91 to 10.65). Given the presence of publication bias, the rectified RR value was 4.43(95%CI:2.32 to 8.12). Owing to the high incidence of malignancies in PM/DM patients, this may result in a large difference between values of OR and RR[50]. From our research, it can be found that the strength of positivity to anti-p155 in DM patients as cancer marker exceeded several times the other identified factors, and accompanying with the establishment of ELISA-based measurement of anti-p155 antibody[51], [52], the detection of anti-p155 was worthy of extensive application in the clinical practice. Casciola-Rosen, Livia et al[53] had demonstrated that immature muscle cells and tumor cells share similarities in their antigenic composition and suggested a paradigm in which auto-antigens are expressed in malignancies and trigger anti-tumor immune responses that may cross-react with autoimmune target tissues, leading to specific clinical phenotypes. The presence of the anti-p155 antibody in the sera of patients with paraneoplastic DM can be further evidence for this hypothesis. The possible role of the anti-p155 antibody as a mediator between DM and malignancy remains to be established.

### Factors associated with a reduced risk of malignancy

Several factors, including ILD, arthritis/arthralgia, Raynaud phenomenon, anti-ENA antibodies and anti-Jo-1 antibody seem to lower the risk for malignancy in PM/DM patients. Identification of these factors may be instructive for the frequency of cancer and for prognosis judgments. Marie and colleagues[54] had recently reported that anti-Jo1-positive patients more commonly developed cancer than those with anti-PL7/PL12 antibodies. Another report published in the 2012 ACR[55] meeting found that the anti-ARS (aminoacyl-tRNA synthetase) antibodies (which include the anti-Jo-1 antibody) were found predominantly among PM/DM patients associated with malignancy. However, neither report met our inclusion criteria. Therefore, whether the presence of the anti-Jo-1 antibody is a protective factor against malignancy in PM/DM patients remains a question for further study. Classically, a negative association between ILD and malignancy in patients with PM/DM was accepted. Intriguingly, the association between ILD and lung cancer had been well recognized for many years[56], [57], [58]. Hill et al[2] found that the incidence of lung cancer in PM/DM patients were significant higher than the general population. Further studies are needed to elucidate that what mechanism makes ILD the protective factor of malignancy in patients with PM/DM.

### Factors that showed inconsistent results (significant or insignificant)

Owing to the different types of research subjects involved and the uncertainty of the relationship between PM/DM and malignancy, we conducted another analysis on studies involving DM patients only when data and resources were sufficient. Analyses of all studies or of studies involving DM patients only yielded results that were sometimes inconsistent for the same factors. Dysphagia was associated with an increased risk of malignancy among PM/DM patients in our meta-analysis, but the results were insignificant in our analysis of studies involving DM patients only. The coefficient associated with the proportion of DM patients in univariate meta-regression analysis was negative, and the number of total DM patients was not small, leading us to speculate that dysphagia is a relevant contributor in increasing the risk for malignancy in PM patients. Other factors – such as ILD, Raynaud phenomenon, arthritis/arthralgia and ESR – were significant when included in an analysis of all studies but insignificant in an analysis of studies involving DM patients only. The aforementioned factors could not be explained this phenomenon. This phenomenon could have occurred because studies involving DM patients only have smaller sample sizes than studies involving PM and DM patients, leading to a larger confidence interval. Additionally, the nature of the association between malignancy and myositis varies. Some population-based studies[30], [59] indicated that the risk of cancer was significantly higher in patients with DM but not in patients with PM. We propose that future studies consider PM patients and DM patients separately to avoid the confounding influence of a possible inconsistent relationship between these two diseases and malignancy.

### Limitations of the study

The limitations of this study should be acknowledged. Firstly, the articles included in this study reported outcomes as dichotomous or continuous. Though some statistical approaches can restate odds ratios as SMDs[60], [61], they are not appropriate for our context because the data in our study do not fit normal or logistic distributions. Application of two separate meta-analyses to dichotomous and continuous outcomes can lead to the decreased power compared to applying a combined meta-analysis. Secondly, there is no uniform definition for cancer-associated myositis (CAM) that may result in differences between the studies. Moreover, in a strict sense, inception cohort data are better adopted for studying the predicting factors/risk factors. In the future research, if conditions allow, it is better to analyze cancer-associated myositis occurring at different time separately. Thirdly, when study the factor of anti-p155, all studies included applied immunoprecipitation assays followed by autoradiography. Since this method is prone to error in identifying the target antigen, such as Mi-2 antibodies will appear in the strip of 155 KD. Nowadays only two studies establish that TIF1-γ is the antigen of p155 in adult patients with DM[51], [52]. Regarding as the same antigen in the eight studies and incorporating their results will also produce certain heterogeneity. Fourthly, in choosing to include crude RR data, we excluded the estimates of adjusted RRs as a result of sparse data. This exclusion may have skewed our results. Fifthly, publication bias and selective reporting are potential limitations but are difficult to assess when the number of studies is small. Our study was based on a comprehensive search of published reports, abstracts and unpublished data presented at conferences, thus reducing possible publication bias. Finally, statistically significant heterogeneity was found in some of the meta-analyses performed, but the pooled estimates should be interpreted cautiously, especially in analyses involving a high degree of heterogeneity (e.g. ESR, CK, LDH, AST, ALT and albumin). Data related to the factors in Table 3 could not be pooled in meta-analysis but were found to associate with increased or reduced risk of malignancy in PM/DM patients and, thus, noteworthy for future studies.

**Table 3:** Summary of data related to factors unable to be pooled in meta-analysis.

Predicting Factor	Study	Year	Description
Constitutional symptoms△	Agne's Sparsa et al[28]	2002	PM/DM patients with constitutional symptoms had a significant higher risk for malignancy (RR,3.32; 95% CI: 1.23 to 8.54).
Targetoid fibers	Nobuo Wakata et al[32]	2002	Histologic changes were observed; targetoid fibers were more common in DM patients with malignancy. (P<0.05).
CA125	Zahir Amoura et al[62]	2005	Tested positive for CA125 was associated with an increased risk of malignancy in PM/DM patients(RR,11.75;95%CI:4.29 to 32.19).
CA19-9	Zahir Amoura et al[62]	2005	Tested positive for CA19-9 was associated with an increased risk of malignancy in PM/DM patients(RR,3.55;95%CI:1.07 to 11.76).
Negative antibody result in routine testing☆	Hector Chinoy et al[41]	2007	DM patients negative for all routine antibodies had a significantly higher risk for malignancy (RR,7.52; 95% CI: 1.03 to 54.92)
Ulcerations and itching	Csilla Andras et al[37]	2008	DM patients with ulcerations and itching had a significantly higher risk for malignancy (RR,3.51; 95% CI: 1.86 to 6.64).
Distal muscle weakness	Csilla Andras et al[37]	2008	DM patients with distal muscle weakness had a significantly higher risk for malignancy (RR,6.19; 95% CI: 3.26 to 11.75).
Herpesvirus infection	Laurence Fardet et al[63]	2009	Dermatomyositis associated with a malignant neoplasmtended to be negatively associated with the risk of herpesvirus infection (HR, 0.16; 95% CI:0.02–1.29).
Lymphocyte count	Laurence Fardet et al[23]	2009	Low baseline lymphocyte count (<1500/mm^3^) was associated with reduced risk of malignancy in DM patients (HR, 0.33; 95% CI: 0.14 to 0.80).
C4	Laurence Fardet et al[23]	2009	A low baseline level of complement factor C4 (<16 mg/L) was associated with increased risk of malignancy in DM patients (HR, 2.74; 95% CI: 1.11 to 6.75).
MSA/MAA-negative*	Trallero-Aragua's et al[6]	2010	A negative result for MSA/MAA had a positive predictive value of 27.8% and a negative predictive value of 89.6% for a diagnosis of CAM.
Rare-infiltrative type muscle pathology	Makoto Uchino et al[64]	2012	The incidence of rare-infiltrative type muscle pathology in DM patients with malignancy was significantly higher than in those without such tumors. (P< 0.05)

△ Constitutional symptoms were defined as weight loss greater than than 5% and/or body temperature above 38°C for at least one week or recurring.

☆ Routine antibodies, including anti-Jo-1, anti-PM-Scl, anti-U1-RNP, anti-U3-RNP and anti-Ku antibodies.

*Myositis-specific autoantibodies, including any antisynthetase autoantibodies (Jo-1, PL-7, PL-12, EJ, OJ, and KS), anti-Mi2 or anti-SRP antibodies. Myositis-associated autoantibodies, including anti-U-RNP, anti-Ku, anti-PM-Scl and anti-Ro.

In conclusion, this is the first comprehensive systematic review and meta-analysis taking into account all presumed predicting factors for malignancy in PM/DM patients. Identifying PM/DM patients who face a high risk for malignancy is important from a public health and clinical perspective as this identification would facilitate early detection of malignancy and, thus, earlier intervention. Developing more complex risk prediction models through these data will be the next logical step. In the meantime, these data provide insight into the etiopathogenesis of CAM.

## Supporting Information

Checklist S1 **PRISMA checklist.**
(DOC)

Table S1 **Characteristics of studies included in meta-analyses.**
(TIF)

Table S2 **Results of methodological quality assessment using the NOS tool.**
(TIF)
