# Supplementary figures and images for: Factors Predicting Malignancy in Patients with Polymyositis and Dermatomyostis: A Systematic Review and Meta-Analysis
Source: PLoS One. 2014 Apr 8;9(4):e94128. doi: 10.1371/journal.pone.0094128 (PMC3979740; doi:10.1371/journal.pone.0094128)

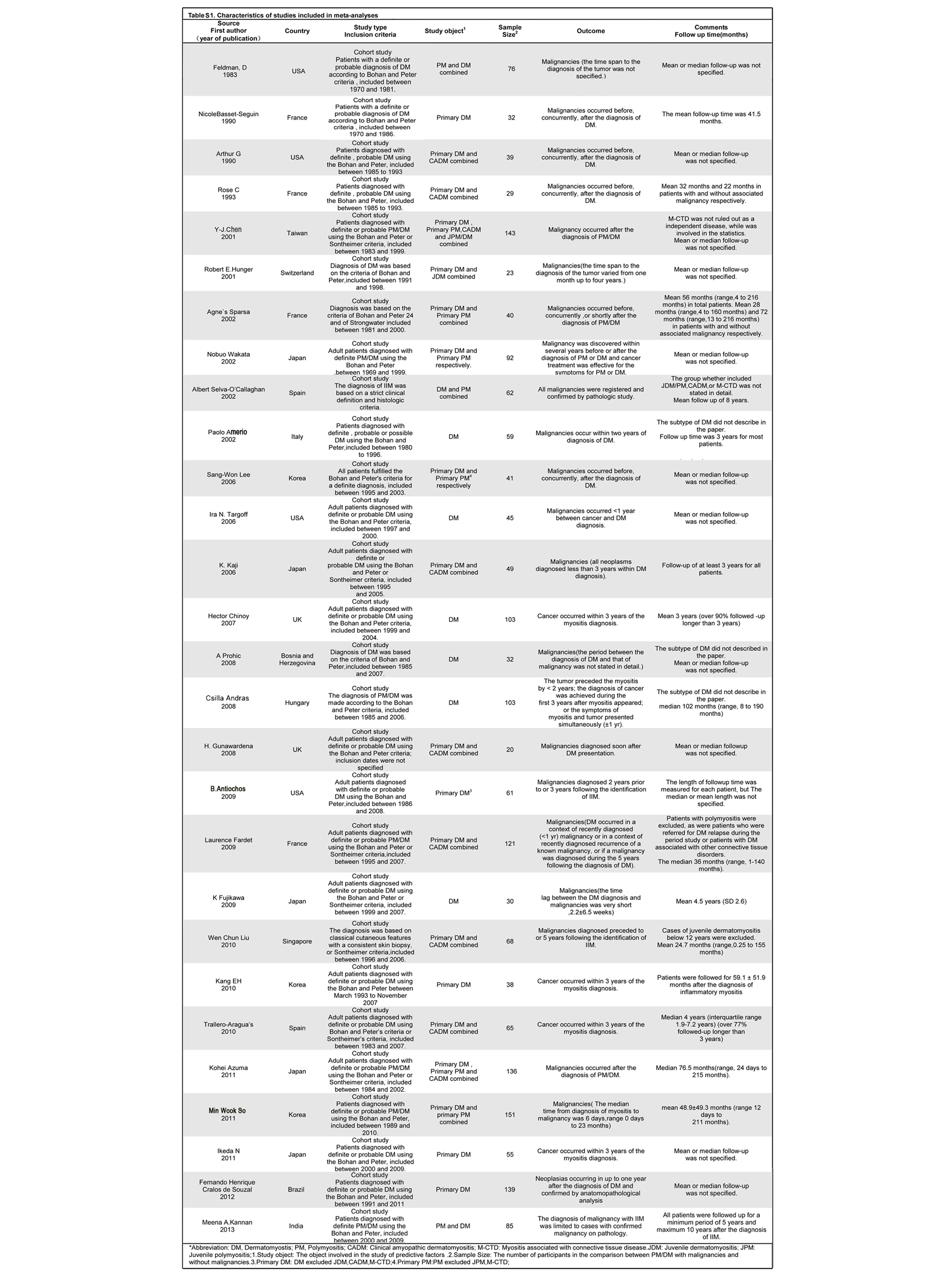

Supplement: Table S1 — Characteristics of studies included in meta-analyses. (TIF) [file pone.0094128.s002.tif]

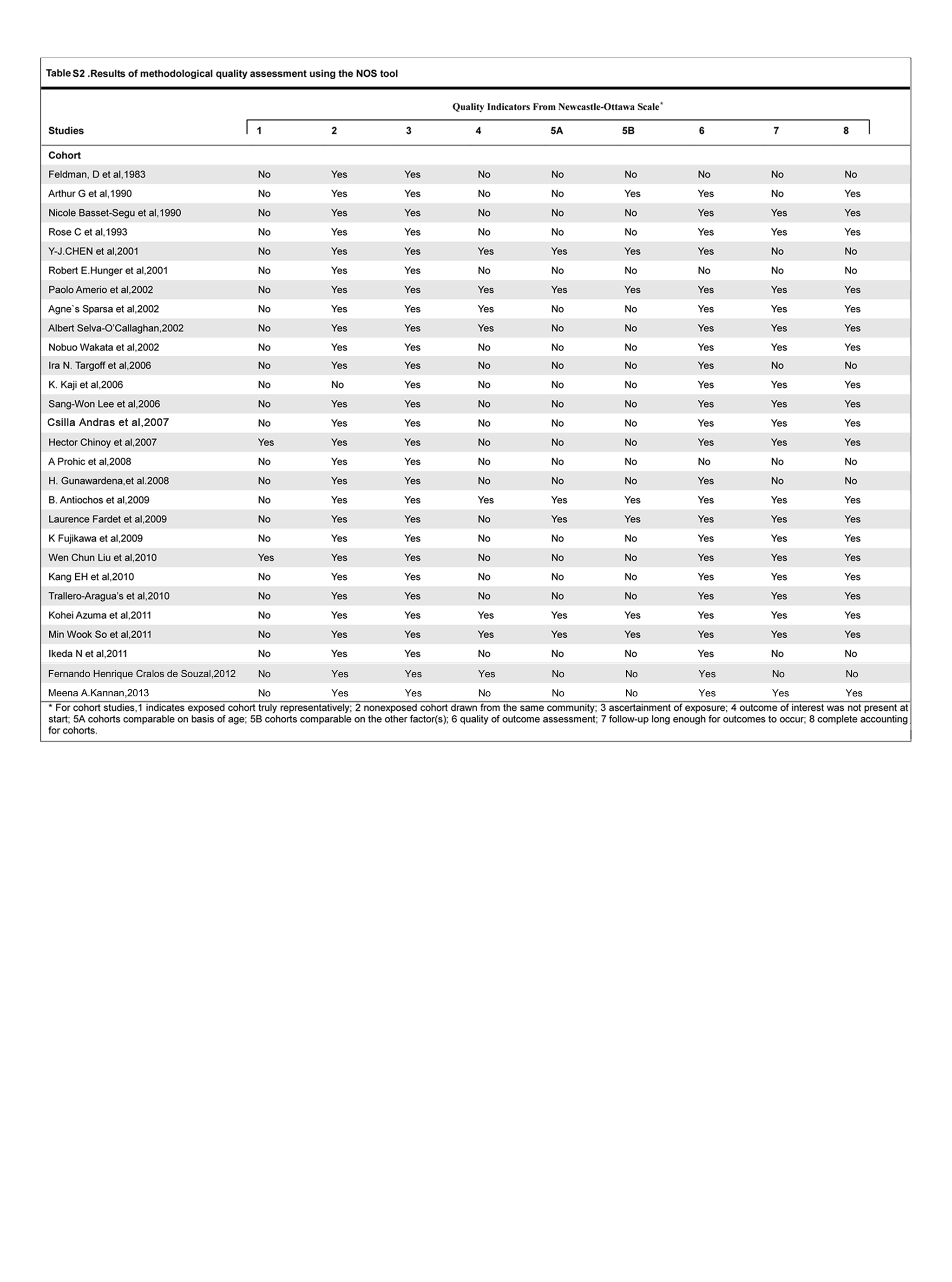

Supplement: Table S2 — Results of methodological quality assessment using the NOS tool. (TIF) [file pone.0094128.s003.tif]
